# Bridge: a GUI package for genetic risk prediction

**DOI:** 10.1186/1471-2156-14-122

**Published:** 2013-12-20

**Authors:** Chengyin Ye, Qing Lu

**Affiliations:** 1 Department of Health Management, School of Medicine, Hangzhou Normal University, Hangzhou, China; 2 Department of Epidemiology and Biostatistics, Michigan State University, B601 West Fee Hall, 909 Fee Road, 48824 East Lansing, MI, USA

**Keywords:** Gene-gene interactions, Optimal receiver operating characteristic curve

## Abstract

**Background:**

Risk prediction models capitalizing on genetic and environmental information hold great promise for individualized disease prediction and prevention. Nevertheless, linking the genetic and environmental risk predictors into a useful risk prediction model remains a great challenge. To facilitate risk prediction analyses, we have developed a graphical user interface package, *Bridge*.

**Results:**

The package is built for both designing and analyzing a risk prediction model. In the design stage, it provides an estimated classification accuracy of the model using essential genetic and environmental information gained from public resources and/or previous studies, and determines the sample size required to verify this accuracy. In the analysis stage, it adopts a robust and powerful algorithm to form the risk prediction model.

**Conclusions:**

The package is developed based on the optimality theory of the likelihood ratio and therefore theoretically could form a model with high performance. It can be used to handle a relatively large number of genetic and environmental predictors, with consideration of their possible interactions, and so is particularly useful for studying risk prediction models for common complex diseases.

## Background

The translation of human genome discoveries into health practice represents one of the major challenges in the coming decades [1,2]. The use of emerging genetic knowledge for early disease prediction, prevention and pharmacogenetics will advance future genomic medicine and lead to more effective prevention and treatment strategies [3]. Among those, disease prediction based on genetic and environmental information is the first step in translating genomics into health [4]. It assesses an individual’s risk of future disease, so that early preventive interventions can be adopted to reduce morbidity and mortality [5]. For this reason, studies to assess the combined role of genetic and environmental information in early disease prediction represent a high priority, as manifested in multiple risk prediction studies now underway [6-12].

The yield from these studies can be enhanced by adopting powerful and computationally efficient study design and analytic tools [13]. We have previously developed an optimal ROC curve (O-ROC) method to quickly evaluate new genetic and environmental findings for potential clinical practice by designing a new risk prediction model, estimating its classification accuracy, and calculating the sample size needed for evaluating the model [14].

If, in the design stage, a proposed risk prediction model appears to be superior to existing models, or if it reaches a desired accuracy level, it may worth developing further for clinical use. To evaluate the risk prediction model on a study sample, we developed a forward ROC curve (F-ROC) method [15]. F-ROC builds on the optimality theory of the likelihood ratio [16], and is thus powerful for risk prediction analysis. It adopts a stepwise selection algorithm to efficiently deal with a large number of predictors and their possible high-order interactions.

To facilitate designing and analyzing risk prediction models, we have implemented the above two methods into the graphical user interface (GUI) software, *Bridge. Bridge* is comprised of two modules, **Test Design** and **Test Build**. The O-ROC approach has been implemented in the **Test Design** module, for designing a risk prediction model. The **Test Design** module uses the essential information (e.g., allele frequencies) of risk predictors from previously published studies or publically available resources to design a risk predictive model, calculating its estimated accuracy and the required sample size to further investigate the model. The F-ROC approach has been built into the **Test Build** module. The **Test Build** module is developed for risk prediction modeling on known risk predictors, as well as for high-dimensional risk prediction based on a large number of potential risk predictors. *Bridge* is freely accessible online at https://www.msu.edu/~qlu/Software.html.

## Implementation

R is open-source software used for statistical computing and graphics. With many built-in statistic functions and excellent scientific graphing capacity, R is now one of the most popularly used statistical software. Although R is widely used in statistics and related fields, it has a limited graphic interface, which makes it difficult for new R users. *Bridge* uses an R graphic user interface (GUI), providing an intuitive and interactive visualization experience for users. Instead of writing code in the R console window, which could be less convenient for new users, the user-friendly interface of *Bridge* allows users to load the datasets and run the program easily by simply clicking either the options from the menu or the buttons from the toolbar. Moreover, for users who prefer to use R console, *Bridge* also provides the access of its functions through R console. In this paper, we give an overview of the package. A detailed description of installation and use of the package can be found in the software vignette.

*Bridge* is comprised of two independent modules, **Test Design** and **Test Build**, for the design and construction of a risk prediction model, respectively. The **Test Design** module serves as a tool for designing a risk prediction study. Given the disease prevalence of a disease of interest and essential information of the known risk predictors (e.g., relative risks) from previous studies and/or public resources, the **Test Design** module plots an estimated receiver operating characteristic (ROC) curve of the proposed predictive model, so that users can easily visualize the estimated discriminating ability of the model. If the model reaches desired level of discriminating ability and worth further investigation, a power analysis can be conducted to make sure sufficient power of the study. Given the power and type I error, the required sample size can be determined by the **Test Design** module to further investigate the proposed model and verify of its classification accuracy.

At least two strategies can be used to select single-nucleotide polymorphisms (SNPs) for designing a risk prediction model. One strategy is to include only disease-susceptibility SNPs that have been replicated in multiple studies and the other is to include as much potentially disease-susceptibility SNPs as possible into the model. Each strategy has its own advantages and disadvantages. Given the limited number of SNPs identified for most of common complex diseases and their small effect sizes, a risk prediction model formed by the former strategy likely has a low AUC value but could have robust performance across different studies. The later strategy could result a risk prediction model with high accuracy, especially when gene-gene interactions exist. Nevertheless, the formed risk prediction model tends to be less stable.

If data is collected to investigate the proposed risk prediction model, we can then use the **Test Build** module of *Bridge* to form and evaluate the proposed model. The **Test Build** module can be used to assess combined effect of known risk predictors (i.e., those identified from previous association studies) in disease prediction, with the consideration of possible high-order interactions. In addition to risk prediction on known risk predictors, the **Test Build** module also allows the users to explore a large ensemble of potential risk predictors and their interactions for improved disease prediction. This strategy is particular useful for complex diseases where a majority of the genetic and environmental risk predictors are unknown. For this strategy, the potentially disease-susceptibility predictors can be chosen based on both biology knowledge and statistical evidence. For instance, we can follow a simple strategy previously used to evaluate different sets of SNPs based on their marginal p-values (i.e., 10^-1^, 10^-2^ ,…, 10^-8^) [8,17]. The **Test Build** module has a built-in forward selection algorithm to handle a large set of predictors. The algorithm is capable of searching for important risk predictors and interactions from a large number of environmental and genetic predictors to further improve the risk prediction model.

In addition, the **Test Build** module has a build-in function for dealing with missing data and provides options for model building and validation (e.g., an option to control the maximum number of risk predictors to be included in the model). The **Test Build** module uses *k*-fold cross-validation to provide internal validation, and can also provide external validation if an independent data is available. The summary results (e.g., the AUC values) for the risk prediction models built on the training and validation datasets are summarized in the *Bridge* output window. Users can also view the proposed model via ROC-curve plots and tree structure plots. The detailed selection process is available under the **Test Build. Results** tab in the output area.

## Results and discussion

We used an empirical study of Crohn’s disease (CD) as an example to illustrate how to use *Bridge* to design and form a risk prediction model.

### Use the Test Design module to design a risk prediction model

**Figure 1:**
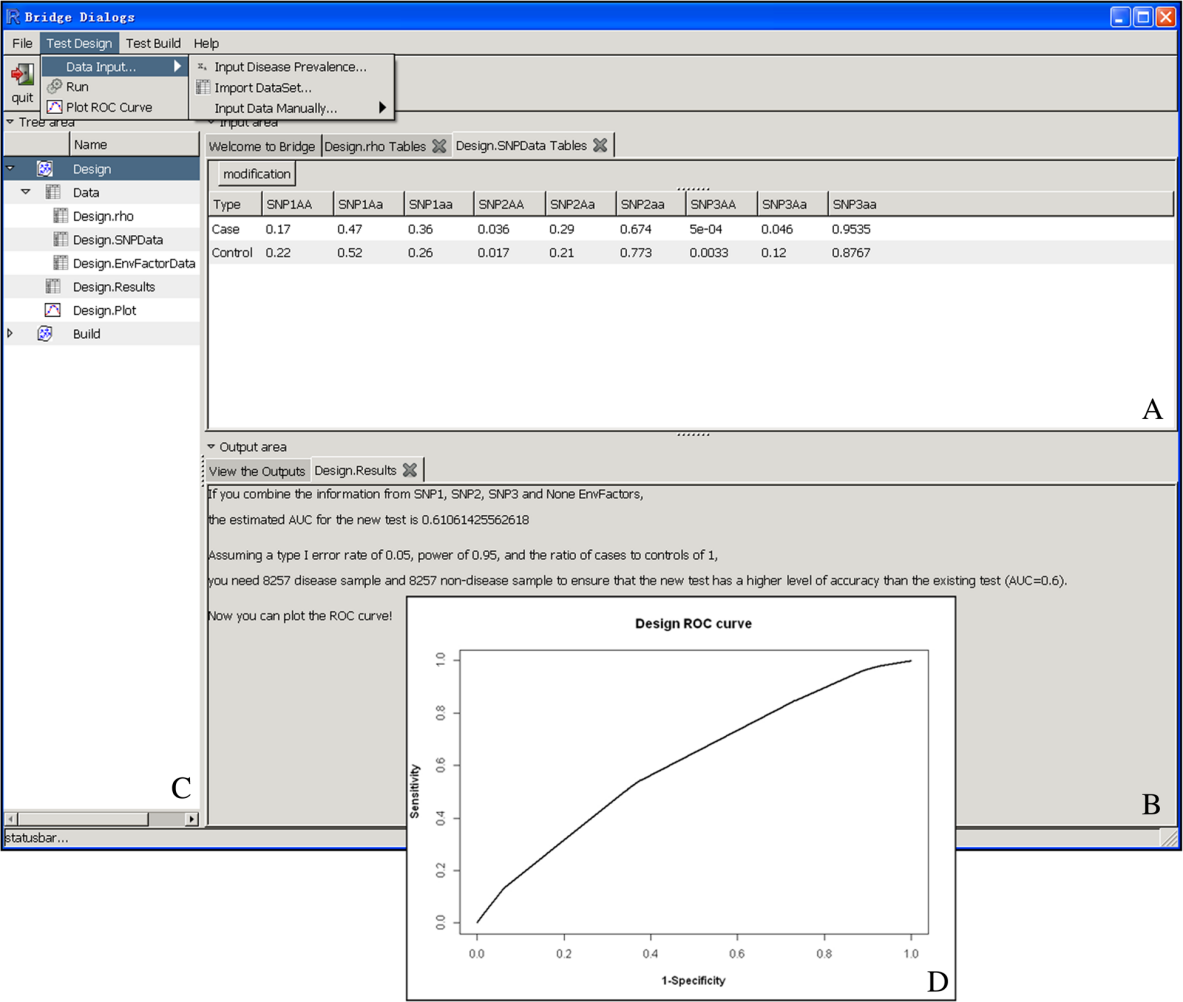
**A screen shot of the Test Design Module. (A)** is the interactive area for entering and viewing data, **(B)** is the **output area** for viewing results, **(C)** is the **tree area** for displaying data and results, and **(D)** is the estimated ROC curve for the CD risk prediction model.

For simplicity, we used three well-replicated CD genetic variants, *rs3828309*, *rs4613763* and *rs11465804*, to design a CD risk prediction model. By using the **Data input** option from the **Test Design** menu, we entered the disease prevalence (ρ = 0.0004) and genotype frequencies of three markers information obtained from a previous study (see Additional file 1). Note that if such information is not available, other information (e.g., relative risk and population frequency) can also be used. By clicking on the **Run** command from the **Test Design** menu, the program estimated that the 3-locus CD risk prediction model had an AUC value of 0.61. Suppose that we are interested in knowing whether the accuracy of the model is significant above the level of 0.60, the **Test Design** module can also calculate the sample size needed to test this hypothesis. Assuming a type I error of 0.05, a power of 0.95, and an equal number of cases and controls, 8257 cases and 8257 controls were required to verify that the proposed model had an AUC value above 0.60. The detailed results related to this analysis were displayed in the **Design Results** tab under the **Output area**. The ROC curve for the estimated risk prediction model could also be viewed by clicking on the **Plot ROC Curve** option from the **Test Design** menu (Figure 1).

### Use the Test Build module to form a risk prediction model

**Figure 2:**
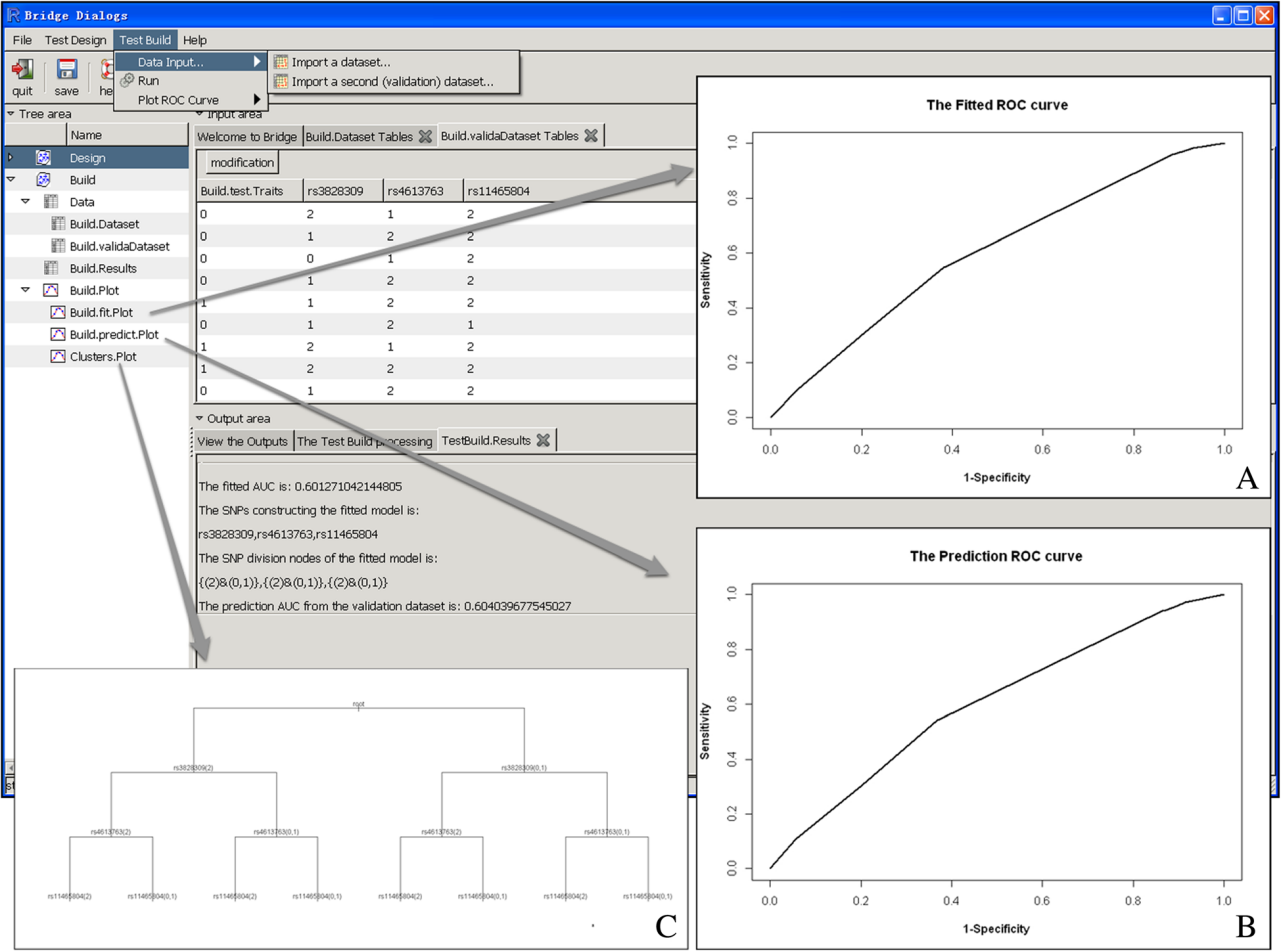
**A screen shot of the Test Build Module. (A)** is the ROC curve of the CD prediction model built on the initial dataset, **(B)** is the ROC curve of the CD model based on the validation dataset, and **(C)** is the tree plot of the risk groups identified by the Test Build module.

In order to further investigate the proposed 3-locus prediction model, we conducted a risk prediction study by using the case–control samples from the Wellcome Trust Crohn’s disease genome-wide association study. From the available 500k SNPs, we selected these 3 CD-related SNPs, *rs3828309*, *rs4613763* and *rs11465804*, and formed a 3-locus model using the **Test Build** module. We first loaded the datasets into the system. The first dataset with 3340 individuals (see Additional file 2) was used for model building, and the second dataset with 1670 individuals was used for model validation (see Additional file 3). With the completion of data loading, samples in both datasets could be viewed in the **Build.Dataset Tables** and **Build.validaDataset Tables** under the **Input area**. Using the samples from the first dataset (i.e., the training samples), we formed a 3-locus model with a fitted AUC value of 0.60. The model was further validated in the second dataset, which attained a predicted AUC value of 0.60. To visualize the formed risk prediction models, the ROC curves could be plotted by using the **Plot ROC curve** option from the **Test Build** menu. The detailed results of the model selection were summarized in the **Test Build. Results** tab under the **Output area**. In the analysis, *rs3828309*, *rs4613763* and *rs11465804* were sequentially entered into the model. In the first step, the module selected *rs3828309,* and split the samples into two distinct risk groups, a high risk group and a low risk group, comprising samples with different genotypes of *rs3828309*. In the sequential steps, it added new markers into the model, and gradually divided samples into more distinct risk groups. The selection process continued until a 3-locus model had been reached. The details of the model building process could be visualized via the tree structure plot under **Clusters.Plot** of **Tree area** (Figure 2). Note that, the risk prediction analysis could also be performed under R console. The detailed description of using the functions in R console could be found in the software vignette.

The above analysis was limited to 3 well-established CD SNPs. In order to consider additional predictors to further improve the 3-locus model, we extended the analysis to 29 potential CD-related SNPs. Using the Wellcome Trust CD dataset (see Additional files 4 and 5), the **Test Build** module identified 5 SNPs and formed a five-locus model with an AUC value of 0.63. The five-locus model was further validated in the testing sample with a predicted AUC value of 0.62. By considering 29 potential CD-related SNPs, the **Test Build** module was able to select 2 additional predictor, *rs3764147* and *rs4263839*, into the model, and further improved the accuracy of the CD risk prediction model.

## Conclusion

With increasing genetic findings from large-scale genetic studies, risk prediction studies are being conducted to evaluate the role of potential genetic and environmental predictors in early disease prediction. While there is increasing interest in such risk prediction research, new bioinformatics tools have not been well developed for this emerging area of research. We developed a GUI package, *Bridge*, to facilitate risk prediction modeling. The software will help an investigator design a study to evaluate a new risk prediction model. It could also be used to form a new risk prediction model based upon multiple genetic and environmental risk predictors, with the consideration of possible interactions. *Bridge* is developed based on a graphical user interface, which can be easily accessed by basic science and clinical researchers.

## Availability and requirements

Project name: Bridge package, Project home page: https://www.msu.edu/~qlu/Software.html. Operating system(s): Linux, Windows, Mac OS X, Programming language: R, Other requirements: R (≥3.0.0), License: GNU GPL, Any restrictions to use by non-academics: none except those posed by the license.

## Abbreviations

ROC curve: Receiver operating characteristic curve; AUC: Area under ROC curve; O-ROC: Optimal ROC curve method; F-ROC: Forward ROC curve method; GUI: Graphical user interface; CD: Crohn’s disease; SNP: Single nucleotide polymorphism.

## Competing interests

The authors declare that they have no competing interests.

## Authors’ contributions

CY and QL participated in the design of the study and implementation of the method. CY drafted the manuscript. QL participated in the conception and design of the study and in editing the manuscript. All authors read and approved the final manuscript.

## Authors’ information

CY: Department of Health Management, Medical School, Hangzhou Normal University, Hangzhou, Zhejiang 310036 P.R. China. Department of Epidemiology and Biostatistics, Michigan State University, East Lansing, MI, 48824 USA, QL: Department of Epidemiology and Biostatistics, Michigan State University, East Lansing, MI, 48824 USA.

## Supplementary Material

Additional file 1 **The example data for the Test Design module.** The data includes three CD-related SNPs, *rs3828309*, *rs4613763* and *rs11465804* and their genotypic frequencies. Click here for file

Additional file 2 **The training data for the Test Build module.** This case–control dataset includes three CD-related SNPs for 3340 individuals, 1323 of which are cases. This dataset was used to build the prediction model. Click here for file

Additional file 3 **The validation data for the Test Build module.** This case–control dataset includes three CD-related SNPs for 1670 individuals, 684 of which are cases. This dataset was used for model validation. Click here for file

Additional file 4 **The second training data for the Test Build module.** This case–control dataset includes 29 CD-related SNPs for 3340 individuals, 1323 of which are cases. This dataset was used to build the prediction model. Click here for file

Additional file 5 **The second validation data for the Test Build module.** This case–control dataset includes 29 CD-related SNPs for 1670 individuals, 684 of which are cases. This dataset was used for model validation. Click here for file
